# Pharmacological Treatment of Secondary Lymphedema

**DOI:** 10.3389/fphar.2022.828513

**Published:** 2022-01-25

**Authors:** Stav Brown, Joseph H. Dayan, Michelle Coriddi, Adana Campbell, Kevin Kuonqui, Jinyeon Shin, Hyeung Ju Park, Babak J. Mehrara, Raghu P. Kataru

**Affiliations:** Plastic and Reconstructive Surgery Service, Department of Surgery, Memorial Sloan Kettering Cancer Center, New York, NY, United States

**Keywords:** lymphedema, tacrolimus, tetracyclines, doxycycline, TH2 cells, CD4+, vascular endothelial growth factor C, VEGF-C

## Abstract

Lymphedema is a chronic disease that results in swelling and decreased function due to abnormal lymphatic fluid clearance and chronic inflammation. In Western countries, lymphedema most commonly develops following an iatrogenic injury to the lymphatic system during cancer treatment. It is estimated that as many as 10 million patients suffer from lymphedema in the United States alone. Current treatments for lymphedema are palliative in nature, relying on compression garments and physical therapy to decrease interstitial fluid accumulation in the affected extremity. However, recent discoveries have increased the hopes of therapeutic interventions that may promote lymphatic regeneration and function. The purpose of this review is to summarize current experimental pharmacological strategies in the treatment of lymphedema.

## Introduction

Lymphedema is a disease that is characterized by progressive fibroadipose tissue deposition resulting from impaired lymphatic drainage. (Rockson, 2001; Kataru et al., 2019; Li et al., 2020a) The pathological changes decrease quality of life (QoL) due to chronic swelling, pain, and loss of function. (Beaulac et al., 2002; Heiney et al., 2007; Warren et al., 2007) Some patients with lymphedema also develop recurrent, sometimes severe, skin infections necessitating hospitalization for intravenous antibiotics and prophylactic antibiotics. (Yuan et al., 2019) Though rare, lymphangitis and sepsis can develop and may result in multiorgan failure and death. These physical and functional manifestations result in higher levels of anxiety and depression and a higher likelihood of chronic pain and fatigue among lymphedema patients. (Stolldorf et al., 2016) The treatment of lymphedema is also very costly and is often not covered by insurance. (Shih et al., 2009; De Vrieze et al., 2020)

Decreased lymphatic drainage capacity leading to lymphedema development can occur due to genetic abnormalities of the lymphatic system or, more commonly, secondary to external insults to the lymphatic system such as infections or surgical injury. (Greene and Goss, 2018) In Western countries, the most common cause of lymphedema is an iatrogenic injury to the lymphatic system during surgical management of solid tumors. (Rockson and Rivera, 2008) Lymphedema develops in approximately 1 in 6 patients treated for melanoma, gynecological or urological tumors, and sarcomas. (Cormier et al., 2010) By far, the most common cause of lymphedema is breast cancer treatment due to the high prevalence of this cancer type. (DiSipio et al., 2013) Although the incidence of breast cancer-related lymphedema (BCRL) is variable depending on the type of study, it is generally thought that approximately 15–40% of all patients who undergo axillary lymph node dissection for breast cancer treatment go on to develop lymphedema. (Cowher et al., 2014; Rockson, 2018; Salinas-Huertas et al., 2021) Patient-related or treatment factors including radiation, taxane chemotherapy agents, and obesity increase the risk of developing secondary lymphedema after cancer surgery. (Shih et al., 2009; DiSipio et al., 2013; Lee et al., 2014; Mehrara and Greene, 2014; Allam et al., 2020) Other studies have suggested that the risk of lymphedema increases with aging and with increasing numbers of lymph nodes removed during surgery. (Guliyeva et al., 2021; Jung et al., 2021) Genetic and sex-linked factors also increase the risk of developing secondary lymphedema. (Finegold et al., 2008; Finegold et al., 2012; Miaskowski et al., 2013) While both primary and secondary lymphedema are more prevalent in women, the role of sex hormones and gender in lymphatic development and function in both physiological and pathological states has yet to be fully deciphered. However, recent studies have increased our understanding of the effects of sex hormones in lymphedema. (Trincot and Caron, 2019; Morfoisse et al., 2021) In addition, sex differences have diverse effects on innate and adaptive immune responses and may also module the risk of lymphedema development. (Klein and Flanagan, 2016)

Despite the high economic and patient costs of lymphedema, current treatment options for most patients with this disease are palliative in nature, focusing on compression garments and manual lymphatic drainage to prevent fluid accumulation rather than addressing the root cause of the disease-lymphatic injury. (Uzkeser et al., 2015; Dayan et al., 2018; Barufi et al., 2021) Lifestyle interventions such as exercise and weight loss are also commonly advocated and can improve the symptoms of the disease but are difficult to maintain; this is an important limitation since lymphedema is a lifelong disease. (Schmitz et al., 2019; Keith et al., 2020) More recently, surgical treatment options have been developed for patients with lymphedema; However, these procedures are not always successful, may not be helpful for patients with severe disease, and can result in complications and morbidity. (Carl et al., 2017; Kayıran et al., 2017; Dayan et al., 2018) Therefore, developing effective, non-invasive treatments for lymphedema that address the pathologic effects of the disease-inflammation, fibrosis, and adipose deposition-is an important goal.

While several reviews have described the potential use of pharmacotherapy agents investigated in clinical studies, the current review aims to summarize both preclinical studies and clinical trials of a wide range of pharmacological strategies to provide a broad view of their potential utility for use in lymphedema. (Badger et al., 2004a; Forte et al., 2019a; Forte et al., 2019b; Forte et al., 2019c; Forte et al., 2019d; Walker et al., 2021) Risk of bias for clinical studies was assessed through the revised Cochrane Risk of Bias tool for randomized controlled trials (RoB2) and Risk of Bias in Non-randomized Studies - of Interventions” (ROBINS-I) tools (Table 1). (Sterne et al., 2016; Sterne et al., 2019)

**TABLE 1:** Summary of Risk of Bias among Randomized (ROB2 tool) and Non-randomized Trials (ROBINS-I tool). A. Risk of Bias for Non-randomized Trials.

Treatment	Study	D1	D2	D3	D4	D5	D6	D7	Overall
Lymphangiogenic interventions (BioBridge™)	Nguyen et al. (2021b)	Moderate	Moderate	Low	Low	Low	Low	Low	Low
Lymphangiogenic interventions (Lymfactin^®^)	Hartiala et al. (2020a)	Moderate	Moderate	Moderate	Low	Low	Moderate	Low	Moderate
TH2 inhibition with neutralizing antibodies	Mehrara et al. (2021)	Moderate	Low	Low	Low	Low	Low	Low	Low

Treatment	Study	D1	D2	D3	D4	D5	Overall
Lymphangiogenic interventions (Lymfactin^®^)	Hartiala et al. (2020b)	Low	No information	High	Low	No information	No information
LTB_4_ Inhibitors	Rockson et al. (2018)	Low	Low	Low	Some concerns	Low	Low
Tetracyclines	Debrah et al. (2006)	Low	Low	Some concerns	Some concerns	Some concerns	Some concerns
Tetracyclines	Mand et al. (2012)	Low	Low	Low	Some concerns	Low	Low

D1: Bias due to confounding.

D2: Bias due to selection of participants.

D3: Bias in classification of interventions.

D4: Bias due to deviations from intended interventions.

D5: Bias due to missing data.

D6: Bias in measurement of outcomes.

D7: Bias in selection of the reported result.

B. Risk of Bias for Randomized Trials.

D1: Bias arising from the randomization process.

D2: Bias due to deviations from intended intervention.

D3: Bias due to missing outcome data.

D4: Bias in measurement of the outcome.

D5: Bias in selection of the reported result.

## Pharmacological Interventions for Secondary Lymphedema

Historically, the pathophysiology of lymphedema was thought to be related to impaired collateral lymphatic vessels formation following surgical injury. (Li et al., 2020b) As a result, many previous studies focused on strategies to improve regeneration of lymphatic vessels using the delivery of lymphangiogenic cytokines. (Li et al., 2020b) However, there is increasing evidence that the pathophysiology of lymphedema is more complex and that increasing lymphangiogenesis alone may not be sufficient to treat the disease. More recent studies have targeted chronic inflammatory reactions in lymphedematous tissues to decrease fibrosis, decrease lymphatic leakiness, improve collateralization, and increase collecting lymphatic pumping.

**FIGURE 1:**
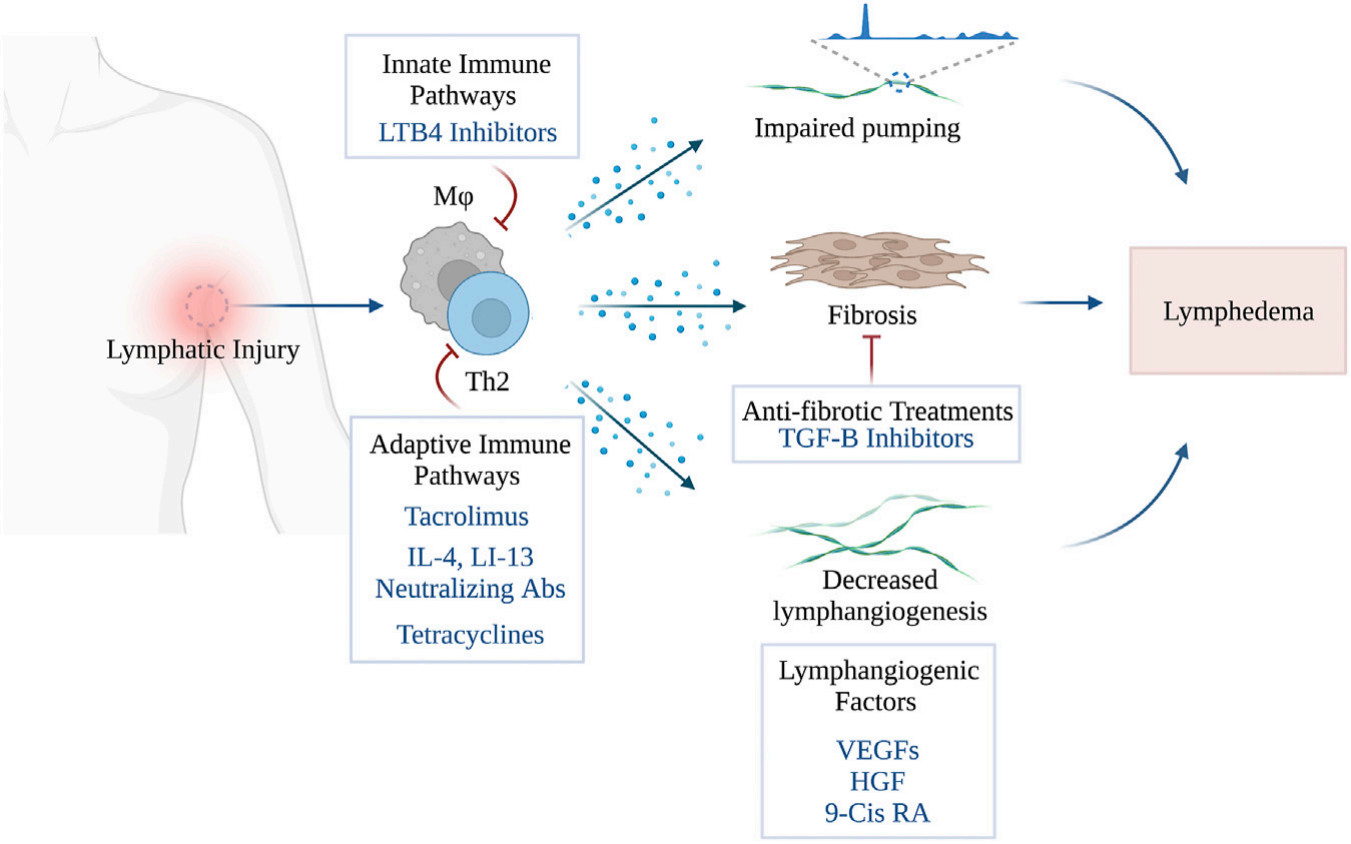
Pharmacological Treatments of Secondary Lymphedema (Created with BioRender). Stav Brown, MD©.

The main therapeutic strategies for the treatment lymphedema consisting of lymphangiogenic interventions, anti-inflammatory treatments and anti-fibrotic agents are summarized in this review (Figure 1). Other treatments for lymphedema have also been reported (e.g., benzopyrones, platelet rich plasma, stem cell therapies), however, these are beyond the scope of the current manuscript and reviewed elsewhere. (Badger et al., 2004b; Forte et al., 2019e; Walker et al., 2021)

## Lymphangiogenic Interventions for Lymphedema

### Vascular Endothelial Growth Factors

VEGFs are a family of growth factors-VEGF-A, VEGF-B, VEGF-C, and VEGF-D-that regulate differentiation, proliferation, survival, and migration of blood and lymphatic endothelial cells (BECs and LECs, respectively). (Oliver, 2004; Olsson et al., 2006) VEGF-C and VEGF-D are high-affinity ligands for the tyrosine kinase receptor VEGFR3, highly expressed in both angiogenic BECs and LECs, and serves as a critical regulator of angiogenesis and lymphangiogenesis. (Oh et al., 1997; Mäkinen et al., 2001a; Mäkinen et al., 2001b; Veikkola et al., 2001; Pytowski et al., 2005; Zarkada et al., 2015; Dayan et al., 2018) Mice with homozygous deletion of *Vegf-c* die *in utero* and have severe lymphatic malformations. (Haiko et al., 2008) Indeed, VEGF-C or VEGF-D activation of VEGFR3 is a key regulator of lymphatic vessel sprouting, LEC proliferation, migration, differentiation, and expression of endothelial nitric oxide synthase (eNOS). (Lahdenranta et al., 2009; Secker and Harvey, 2021) Heterozygous *de novo* and inherited *VEGFR3* mutations have been identified in a congenital or infantile-onset form of lymphedema known as Milroy disease. (Ghalamkarpour et al., 2009; Connell et al., 2013; Online Mendelian Inheritance in Man, 2021) Transgenic mice that express a soluble (i.e., inactivating) form of VEGFR3 have impaired fetal lymphangiogenesis, regression of lymphatic vessels, and a lymphedema-like phenotype including hindlimb swelling and dermal fibrosis. (Mäkinen et al., 2001a)

The observation that treating transgenic mice with a heterozygous inactivating *Vegfr-3* mutation—a mouse model of primary lymphedema—using virus-mediated VEGF-C gene therapy increased lymphangiogenesis and formation of functional lymphatics led to the hypothesis that these treatments may also be effective for the treatment of secondary lymphedema. (Karkkainen et al., 2001) This hypothesis was supported by follow-up studies in which recombinant VEGF-C was used in animal models of secondary lymphedema. For example, Szuba and colleagues showed that a single 100 μg dose of recombinant human VEGF-C into the surgical bed of a rabbit ear model of lymphedema resulted in histologic evidence of lymphangiogenesis, decreased dermal hypercellularity, and improvements in Indocyanine Green (ICG)-based quantifications of lymphatic function compared to controls. (Szuba et al., 2002) In follow-up studies, this group also showed that delivery of recombinant human VEGF-C improved outcomes in a mouse tail model of lymphedema by increasing lymphangiogenesis, improving the morphology of cutaneous lymphatic vessels, and the ability of lymphatic channels to transport immune cells. (Cheung et al., 2006; Jin et al., 2009)

Topical formulations of VEGF-C have been developed to avoid direct injections of the growth factor and to elicit sustained release. These studies have used acidic gelatin hydrogels to generate a sustained release of biologically active VEGF-C. (Hwang et al., 2011) Combining VEGF-C hydrogel with extracorporeal shock wave therapy or adipose-derived stem cells (ADSCs) was even more effective in reducing edema and increasing lymphangiogenesis. (Kim et al., 2013) Indeed, ADSCs alone have been shown to increase lymphatic regeneration and acquire a lymphatic endothelial phenotype in a mouse tail model of lymphedema. (Conrad et al., 2009) Other studies have shown that ADSCs increase lymphangiogenesis by secreting lymphangiogenic growth factors. (Yan et al., 2011; Ahmadzadeh et al., 2020)

Other groups have incorporated recombinant VEGF-C in nanofibrillar collagen scaffolds (BioBridge™) designed to guide lymphangiogenesis across an area of obstruction. (Hadamitzky et al., 2016) Using a porcine model, control animals that underwent lymphatic ligation were compared with animals treated with BioBridge alone or with BioBridge™ with autologous lymph node transplantation. Three months following surgery, animals treated with BioBridge™, with or without lymph node transplantation, had decreased bioimpedance ratio—a measure of fluid accumulation in the skin—suggesting that this treatment increased functional lymphatic regeneration. In follow-up studies using a rat lymphedema model, the authors compared control animals that had undergone lymphadenectomy with those implanted with BioBridge™ with or without autologous ADSCs. Four months after surgery, animals treated with BioBridge™ (with or without ADSCs) had decreased swelling and increased lymphatic collateralization towards other lymph node drainage basins. (Nguyen et al., 2021a) The efficacy of the device was also retrospectively reviewed following implantation in patients treated with scar release and vascularized lymph node transplantation (VLNT) or lymphovenous anastomosis (LVA) for treatment of secondary lymphedema of the upper or lower extremity. (Danielle et al., 2019) ICG fluorescence lymphography in a patient treated with VLNT and BioBridge demonstrated qualitatively decreased dermal backflow at the site of device implantation and dynamic uptake of ICG in the newly formed collectors draining into the area where VLNT was performed. The authors noted complete or nearly complete edema reduction in two patients with mild lower extremity lymphedema treated with BioBridge implantation and LVA and one patient with a moderate disease of the upper extremity treated with BioBridge™ and VLNT. These interesting results were followed up by a retrospective cohort study of 29 patients with stage I-III lymphedema of the upper or lower extremity treated with LVA or VLNT with (*n* = 18 patients) or without (*n* = 11 patient) BioBridge™ implantation. (Nguyen et al., 2021b) At 1-year follow-up, the authors reported significantly decreased edema in patients treated with LVA or VLNT and implanted with BioBridge™ as compared to patients treated with surgery alone (although the patients treated with LVA or VLNT also showed improvements). Lymphatic mapping with ICG quantitatively showed more collectors and decreased dermal backflow in patients implanted with BioBridge. These findings are very exciting and require further study in a randomized control trial.

Recombinant VEGF-C is expensive, and its use in large series of animal experiments is cost-prohibitive. As a result, some authors have utilized gene therapy methods to increase VEGF-C expression in lymphedema models. Local delivery of naked plasmid VEGF-C DNA in the rabbit ear or mouse tail models increases VEGFR3 expression, decreases swelling and fibroadipose tissue deposition, and improves lymphatic function as assessed using lymphoscintigraphy. (Yoon et al., 2003) Similar results were noted by Liu *et al.* using a rat hindlimb model of secondary lymphedema. (Liu et al., 2008)

The low efficiency of naked plasmid technique led other groups to develop viral vectors for VEGF-C delivery. Saaristo *et al.* developed a VEGFR3 specific VEGF-C isoform (VEGF-C156S) since VEGF-C activation of VEGFR2 results in blood vessel proliferation and tissue edema. (Saaristo et al., 2002) Delivery of VEGF-C156S using an adenoviral vector to a mouse model of primary lymphedema improved lymphangiogenesis without blood vascular effects. These observations led to the development of adeno-associated viral vectors (AAV) for VEGF-C delivery since AAV vectors elicit decreased host inflammatory responses and mediate longer-term delivery of the construct compared with adenoviral vectors. (Lai et al., 2002)

Administration of AAV-VEGF-C and VEGF-C vectors to the site of surgical injury increases regeneration and differentiation of lymphatic vessels after lymph node dissection in porcine and mouse models. (Tammela et al., 2007; Lähteenvuo et al., 2011) Similarly, delivery of adenoviral VEGF-C either in the lymph node or in the tissues surrounding it increases regrowth of lymphatic vessels and preserves the architecture of the lymph node compared with controls. (Honkonen et al., 2013) These findings suggest that VEGF-C delivery may be helpful for patients treated with autologous VLNT—a surgical treatment for lymphedema. This hypothesis is further supported by other studies demonstrating that lymphatic regeneration following VLNT is associated with increased VEGF-C expression and that injections of recombinant VEGF-C increase lymphatic regeneration and reconnection in autologous transplanted lymph node fragments. (Sommer et al., 2012; Schindewolffs et al., 2014; Huang et al., 2016)

Positive results with preclinical models of VEGF-C delivery in combination with VLNT led to human phase I and phase II clinical trials; Lymfactin^®^ is an investigational adenoviral type 5-based gene therapy vector that expresses human VEGF-C. (Hartiala et al., 2020a) A phase I clinical trial reported the use of Lymfactin^®^ in 15 patients with breast cancer-related lymphedema who underwent lymph node transplantation and lymfactin injections of various doses (NCT02994771). The authors reported no dose-limiting toxicities and, at 1-year follow-up, they noted that the drug was well tolerated. Two patients developed infections in the lymphedema limb requiring hospitalization; however, infections occur commonly with lymphedema and could not be definitively attributable to the drug. A follow-up phase 2 double-blind, randomized, placebo-controlled, multicenter clinical trial with 39 patients with 12-months follow-up was reported as accruing patients in an abstract published in Cancer Research Communications (NCT03658967) (Hartiala et al., 2020b); however, a final report has not been published in peer-reviewed journals. The study was inconclusive in a company press report. (Herantis Pharma Plc, 2021) Nevertheless, the drug was generally safe and well-tolerated. It is unclear if the company plans to continue drug development.

The advent of mRNA-based gene delivery techniques has garnered significant attention recently due to the success of novel COVID-19 vaccines utilizing this approach. These vaccines use nucleoside-modified mRNA encapsulated in lipid nanoparticles (LNPs) and have been shown to be safe and effective in various preclinical models. (Pardi et al., 2018; Magadum et al., 2019; Sahu et al., 2019; Polack et al., 2020; Baden et al., 2021; Skowronski and De Serres, 2021; Wang, 2021) Nucleoside-modified mRNA have several advantages to adenovirus and adeonoassociated virus vectors, including lack of potential integration into the host genome, no pre-existing anti-vector immunity, the potential for repeated treatments without inflammatory or immune sequela, controlled and transient protein production, and decreased manufacturing costs. (Brown et al., 2020)

Szoke *et al.* recently reported on the use of nucleoside modified VEGF-C mRNA delivered using lipid nanoparticles to promote organ-specific lymphangiogenesis and treat experimentally induced lymphedema. (Szőke et al., 2021) They showed that injection of the VEGF lipid nanoparticles increased VEGF-C expression locally for as long as 20 days after treatment. This increased VEGF-C production significantly increased lymphangiogenesis that persisted even 60 days after injection. Importantly, the authors showed that intradermal injections did not induce significant blood vessel proliferation locally or in other organ systems, induce immune response activation, or have off-target effects in the lungs or small intestines. This is important since adenoviruses and AAVs can increase gene expression in other organ systems after systemic absorption. Most importantly, the authors reported that a single VEGF-C mRNA lipid nanoparticle injection significantly increased edema formation and adipose deposition in a mouse hindlimb model of lymphedema.

Although VEGF-C treatments have shown potential for clinical application, several lines of experimental and clinical evidence suggest that a cautious approach is needed. One interesting finding is that the expression of VEGF-C is increased in lymphedematous tissues (and serum) and may contribute to increased interstitial fluid accumulation. (Jensen et al., 2015) Thus, it is unclear why increasing the concentration of a cytokine that is already increased in lymphedematous tissues may be a clinically effective means of treating lymphedema. The reasons for this paradoxical increase in VEGFC remain unknown. However, based on our observations in preclinical models, our group has suggested that the increased concentration of VEGF-C is counterbalanced by increased expression of anti-lymphangiogenic cytokines (e.g., TGF-B1, interferon gamma, IL4, IL13) that decrease lymphatic endothelial proliferation and increase lymphatic leakiness. (Kataru et al., 2011; Gardenier et al., 2017; Gousopoulos et al., 2017; Park et al., 2018) Anti-lymphangiogenic cytokines can directly decrease responsiveness of lymphatic endothelial cells to VEGF-C thus decreasing the efficacy of exogenous VEGF-C delivery for the treatment of lymphedema. (Savetsky et al., 2015; Shin et al., 2015; Ogata et al., 2016) This hypothesis is supported by the finding that VEGF-C overexpression induces transient lymphatic hyperplasia but does not improve lymphatic function in the mouse tail model of lymphedema. (Goldman et al., 2005) It is also possible that over-expression of VEGF-C increases inflammation and blood vessel leakiness thus contributing to increased swelling. (Gousopoulos et al., 2017) Taken together, these findings suggest that the pathophysiology of lymphedema is more complex than a simple deficiency of VEGF-C (or other lymphangiogenic cytokines) and may explain why surgical interventions that decrease scarring or improve lymphatic function are necessary as adjuncts to exogenous VEGF-C delivery for optimal results.

Another potential concern regarding VEGF-C therapy for patients with lymphedema or those at risk of developing the disease, is that VEGF-C is a key regulator of tumor cell growth and metastasis in a variety of solid tumors, including breast cancer. (Skobe et al., 2001; Chen et al., 2012; Kong et al., 2021) Increased VEGF-C expression increases regional lymph node and distant metastasis and is associated with decreased overall prognosis. (Hoshida et al., 2006; Lohela et al., 2009; Tammela and Alitalo, 2010) VEGF-C also promotes proliferation, migration, and invasion of epithelial breast cancer cells. (Karkkainen et al., 2001; Kong et al., 2021) Thus, the delivery of large doses of VEGF-C at the time of surgery to prevent lymphedema development may impact the oncologic aspects of patient care and requires careful study.

### Other Lymphangiogenic Growth Factors

Other lymphangiogenic growth factors have also been used in preclinical models. For example, fibroblast growth factor 2 (FGF2), promotes lymphangiogenesis by inducing the expression of VEGF-C and VEGF-D. (Javerzat et al., 2002) Onishi *et al.* demonstrated that topical FGF2 reduces edema, increases lymphatic vessel density, improves lymphatic function (evaluated as the fluorescence intensity of indocyanine green every 3 days), and upregulates VEGF-C expression in a rat tail model of lymphedema. (Onishi et al., 2014)

Hepatocyte growth factor (HGF) regulates migration, proliferation, and differentiation of a wide variety of cells. (Nakamura et al., 1989) HGF is also a powerful promoter of lymphangiogenesis and can act directly on LECs by interacting with its high-affinity receptor-mesenchymal-epithelial transition factor (c-MET). (Kajiya et al., 2005; Cao et al., 2006; Saito et al., 2006) *HGF/MET* mutations have been identified in patients with primary lymphedema, and single nucleotide polymorphisms of this gene are associated with an increased risk of developing BCRL. (Finegold et al., 2008) *MET* somatic activating mutations have also been identified in lymphovenous malformations. (Palmieri et al., 2021) Experimental studies have also shown that HGF may have some utility in treating lymphedema. For example, weekly HGF gene therapy decreased swelling and increased lymphangiogenesis in a rat tail model of lymphedema. (Saito et al., 2006)

Retinoic acid agonists such as 9-cis retinoic acid (RA)--a metabolite of vitamin A that is FDA-approved for treating Kaposi’s sarcoma and chronic eczema--increase migration and differentiation of LECs and lymphangiogenesis *in vitro* and preclinical models of lymphedema. (Choi et al., 2012; Wong, 2021) 9-cis RA treatment also effectively prevented the development of postsurgical lymphedema in a mouse hindlimb model of lymphedema by promoting the formation of collateral lymphatics by activating FGFR signaling. (Jin et al., 2012; Bramos et al., 2016; Perrault et al., 2019; Daneshgaran et al., 2020)

Despite the exciting developments in bench-to-bedside research throughout the past decade, it is important to address the gaps in current clinical trials, including relatively small patient cohorts and lack of standardized and reproducible outcome measures. These challenges and shortcomings are nicely reviewed in other manuscripts. (Forte et al., 2019f; Herantis Pharma Plc, 2021; Walker et al., 2021)

## Anti-Inflammatory Treatments for Lymphedema

### Innate Immune Pathways

Inflammation is a clinical hallmark of lymphedema and a consistent finding in clinical specimens and preclinical models. However, the importance of inflammation to the pathophysiology of lymphedema has only recently been appreciated. Studies by Rockson’s group highlighted the key role of inflammatory cells by showing that anti-inflammatory treatments were highly effective in preclinical models; In an important study, Dr. Rockson’s group showed that lymphatic insufficiency in a mouse model of lymphedema resulted in a marked inflammatory response in the skin with a molecular signature of acute inflammation, fibrosis, and oxidative stress. (Tabibiazar et al., 2006) These studies led to an investigation on the effects of ketoprofen, a non-steroidal anti-inflammatory (NSAID) medication, in a mouse tail model of lymphedema. (Nakamura et al., 2009; Tian et al., 2017; Rockson et al., 2018) Ketoprofen is indicated to manage pain, dysmenorrhea, rheumatoid arthritis, and osteoarthritis. (Kawai et al., 2010; U.S. Prescribing information, 2021a) While it has been shown to have inhibitory effects on prostaglandin and leukotriene synthesis as well as anti-bradykinin activity, its mode of action, like that of other NSAIDs, has not been fully elucidated. (U.S. Prescribing information, 2021a)

Using a mouse-tail model, Nakamura *et al.* demonstrated that subcutaneous injection of ketoprofen starting on postoperative day 3 (POD 3) reduced tail volumes and epidermal thickness, decreased inflammation, and improved the histologic changes of the capillary lymphatics. (Nakamura et al., 2009) While ketoprofen treatment had broad anti-inflammatory effects, the authors also observed a paradoxical increase in the expression of inflammatory cytokines TNF-α and MCP-1, along with robust up-regulation of VEGF-C and VEGFR-3. The authors hypothesized that ketoprofen promoted lymphangiogenesis via TNF-α mediated VEGF-C up-regulation. (Nakamura et al., 2009)

The promising results of these preclinical studies have led to a clinical trial using ketoprofen in patients with either primary or secondary lymphedema of the upper or lower extremity (NCT02257970). (Rockson et al., 2018) In an exploratory portion of the trial, 21 patients with lymphedema were enrolled in an open-label trial receiving ketoprofen 75 mg by mouth 3 times daily for 4 months (Rockson et al., 2018) Ketoprofen therapy significantly improved skin pathology as evidenced by decreased dermal thickness, collagen deposition, co intercellular mucin deposition, and perivascular inflammation compared with baseline biopsies before initiation of treatment. (Rockson et al., 2018) However, no significant differences in either limb volume or bioimpedance were demonstrated following ketoprofen treatment. (Rockson et al., 2018) In the second phase of the study, 34 patients (16 treated with ketoprofen 18 without) with the same inclusion criteria were recruited for a randomized, placebo-controlled trial. This portion of the study again demonstrated improvements in skin histology as evidenced by decreased skin thickness and decreased plasma granulocyte CSF (G-CSF) expression following Ketoprofen administration. (Rockson et al., 2018) However, treatment with ketoprofen did not significantly decrease limb volumes or bioimpedance. In addition, while treatment with ketoprofen in this study was reported to be safe with no serious adverse events, it is important to note that prolonged NSAID use is limited by a black box warning regarding cardiovascular, renal, and gastrointestinal toxicity. (FDA, 2021; U.S. Prescribing information, 2021a)

Unlike other commonly used NSAIDs, ketoprofen inhibits both the 5-lipoxygenase (5-LOX) and the cyclooxygenase (COX) pathways. (Nakamura et al., 2009) Therefore, to determine which pathway was important for the beneficial effects of ketoprofen on lymphedema, Dr. Rockson’s group used a mouse model of lymphedema to analyze the efficacy of specific inhibitors of each pathway and found that the therapeutic effect of ketoprofen was attributable to the inhibition of the 5-LOX pathway metabolite leukotriene B4 (LTB_4_). (Tian et al., 2017) Inhibition of LTB_4_ with Bestatin (*Ubenimex*), a selective LTB_4_ antagonist with no anti-COX activity, decreased swelling, and increased lymphatic function in the mouse tail model of lymphedema. Mechanistically, LTB_4_ had a bimodal effect on lymphangiogenesis: low doses of LTB_4_ increased lymphangiogenesis while high concentrations—as found in lymphedema—inhibited lymphangiogenesis. These encouraging results have led to a multicenter placebo-controlled trial of 46 lower extremity lymphedema participants who received 150 mg bestatin 3 times daily for 6 months (NCT02700529). (ULTRA, 2021) The results of this trial are currently pending.

Interestingly, a recent study utilizing the mouse tail model of lymphedema showed that although treatment with bestatin increased lymphatic contractile activity, drug therapy did not modulate the overall leukocyte populations in the draining lymph nodes and did not decrease tail swelling. (Cribb et al., 2021) The authors suggested that addressing lymphatic vessel dysfunction is insufficient for lymphedema prevention/treatment.

### Adaptive Immune Pathways

Recent studies from our lab and others have shown that CD4^+^ T helper (TH) cells in lymphedematous tissues play a key role in the pathophysiology of lymphedema. Using biopsy specimens from women with unilateral BCRL and mouse models of lymphedema, we have shown that the number of infiltrating TH cells is increased in lymphedematous tissues. Further, we found that increasing numbers of CD4^+^ cells are correlated with the clinical severity of the disease. (Avraham et al., 2013) Transgenic mice lacking CD4^+^ cells or mice treated with neutralizing antibodies against CD4^+^ cells did not develop lymphedema following lymphatic injury in the mouse tail model of the disease. In contrast, depletion of other inflammatory cell types such as B cells, cytotoxic (CD8^+^) T cells, or macrophages either has no effect or worsens the pathology. (Zampell et al., 2012a; Ghanta et al., 2015; García Nores et al., 2018) Indeed, even limited numbers of CD4^+^ cells that remain after total body irradiation or adoptive transfer of CD4^+^ cells to CD4 knockout mice resulted in the development of lymphedema following skin and lymphatic resection. (Ly et al., 2019a)

### Tacrolimus

Preclinical mouse lymphedema models have shown promising results when T cell infiltration and activation in the skin are inhibited using topical medications. (Forte et al., 2019b) Indeed, these pathologic changes share distinct similarities to other chronic skin inflammatory diseases such as atopic dermatitis that are treated with topical T cell inhibitors and steroids. (Brandt and Sivaprasad, 2011; James and Kwok, 2011; Su et al., 2017; Acevedo et al., 2020) *Tacrolimus*, a macrolide calcineurin inhibitor, exerts its anti-T-cell properties by inhibiting the Nuclear factor of activated T-cells (NFAT) signaling, ultimately decreasing IL-2 expression. Because IL-2 is essential for both T-cell activation differentiation, and proliferation, Tacrolimus administration results in profound CD4^+^ cell immunosuppressive effects. (Smith, 1988; Clipstone and Crabtree, 1992; Chow et al., 1999; Rautajoki et al., 2008; Liao et al., 2013) The topical formulation is FDA-approved for treating cutaneous inflammatory conditions, including atopic dermatitis, psoriasis, and localized scleroderma. (Ruzicka et al., 1997; Mancuso and Berdondini, 2005; Wang and Lin, 2014; U.S. Prescribing information, 2021b) Topical treatment of mouse tails either before lymphedema development or once it had become established was highly effective for reducing fibroadipose tissue deposition and improving lymphatic function. (Gardenier et al., 2017) This treatment significantly decreased macrophage and T cell infiltration and expression of inflammatory cytokines. Importantly, topical treatments did not result in significant systemic absorption, thus mitigating the potentially toxic effects of this drug on generalized immune responses and kidney function. This concept is supported by other studies demonstrating that intermittent topical tacrolimus application for up to 1 year does not result in significant blood concentrations of the drug. (U.S. Prescribing information, 2021b) Clinical studies with periodic blood sampling in a total of 1,391 patients have confirmed this finding, demonstrating a measured blood concentration of less than 2 ng/ml in 90% of patients and 30-fold less exposure of Tacrolimus as compared with oral administration. (U.S. Prescribing information, 2021b) Nevertheless, our group has taken a cautious approach to using Tacrolimus clinically since application of the drug to a large body surface area (e.g., an entire arm or leg) as would occur in patients with lymphedema may result in systemic absorption.

### TH2 Inhibition with Neutralizing Antibodies

Naïve TH cell differentiation requires interaction and activation by antigen-presenting cells (APCs) in regional lymph nodes. Using adoptive transfer experiments with tagged leukocytes, our lab has shown that lymphatic ligation results in rapid activation of APCs in the skin distal to the zone of injury within 12 h. These APCs migrate to regional draining lymph nodes where they interact with naïve CD4^+^ cells to promote a mixed TH1/TH2 inflammatory response and are then released into the systemic circulation. (García Nores et al., 2018) Inhibition of T cell release by the lymph node using FTY 720 (Fingolimod^®^)-a sphingosine 1-phosphate receptor 1 agonist that is FDA approved for the treatment of multiple sclerosis--prevents lymphedema development in the mouse tail model of lymphedema, suggesting that T cell differentiation in the lymph node plays a key role in the pathophysiology of the disease. (García Nores et al., 2018; U.S. Prescribing information, 2021) Activated TH cells migrate back to the lymphedematous tissues via the expression of cell surface receptors that recognize their cognate ligands on inflamed blood vessels in the lymphedematous tissues. (García Nores et al., 2018)

TH cells can differentiate into various lineages such as TH1, TH2, TH17, and T-regulatory cells. (Zhu et al., 2010) We have found that TH2 cell differentiation is a key process in the pathophysiology of lymphedema. (Ly et al., 2019b) This concept is supported by the fact that transgenic mice with impaired TH2 differentiation potential (Stat-6 knockouts) do not develop lymphedema following skin/lymphatic ablation. In contrast, mice with impaired TH1 differentiation potential (T-bet knockouts) develop lymphedema indistinguishable from wild-type controls. (Ly et al., 2019b) Similarly, neutralizing antibody inhibition of TH2 differentiation using interleukin 4 (IL4) or IL13—cytokines necessary for differentiation of naïve CD4^+^ cells to the TH2 lineage—is effective in both treatment and prevention of lymphedema in the mouse tail model. Mice treated in this manner have decreased fibroadipose tissue deposition, decreased inflammation, improved lymphatic collecting vessel pumping capacity, decreased lymphatic leakiness, and overall improved lymphatic function. (Zampell et al., 2012a; Avraham et al., 2013) In addition to causing extracellular matrix deposition, T cell-derived cytokines directly affect LECs, decreasing their responsiveness to lymphangiogenic growth factors and actively inhibiting lymphangiogenesis. (Savetsky et al., 2015; Shin et al., 2015; Gardenier et al., 2017) These findings may provide a mechanistic rationale for the finding that the expression of VEGF-C is upregulated in lymphedematous tissues, yet the formation of functional collateral lymphatics is impaired in patients with lymphedema.

Our observations that inhibition of TH2 differentiation improved lymphedema outcomes in preclinical models led to a clinical trial to study the efficacy of IL4/IL13 neutralizing antibodies for the treatment of unilateral BCRL (NCT02494206). (Mehrara et al., 2021) Neutralizing antibodies are now commonly used to treat many chronic inflammatory diseases due to the decreased toxicity of these targeted treatments compared with traditional treatments. (Hansel et al., 2010) We recruited 9 women with stage I/II BCRL to a phase I, open-label trial utilizing QBX258, an experimental drug consisting of two humanized monoclonal antibodies that inhibit IL4 (VAK296) and IL13 (QAX576). (Mehrara et al., 2021) Patients that met the inclusion criteria were treated with once-monthly intravenous infusions of QBX258 for 4 months. (Mehrara et al., 2021) Outcomes were analyzed immediately after and 4 months following cessation of treatment. QBX258 treatment was safe, and most adverse events were minor and self-limited. Treatment with QBX258 improved QoL measurements, skin stiffness, and histologic changes in the lymphedematous limb. Drug treatment significantly decreased keratinocyte hyperplasia, mast cell infiltration, and the expression of TH2 inducing cytokines in the skin. However, similar to the results of the ketoprofen trials, we found no significant improvements in arm volumes or bioimpedance. Future studies with larger patient populations and more effective IL4/IL13 inhibiting compounds (e.g., Dupilmab) are needed.

### TH2 Inhibition with Tetracyclines

Tetracycle antibiotics have shown promise for treating filariasis, a parasitic disease that results in the development of lymphedema due to chronic lymphatic obstruction. (Mand et al., 2012; Debrah et al., 2006; U.S. Prescribing information, 2021c) The lymphatic pathology in filariasis is primarily host-immune mediated, and lymphedema severity is closely related to the magnitude of CD4^+^ T cell immune responses. (Babu et al., 2009; Babu and Nutman, 2014) Filarial infections also increase plasma levels of VEGF-C and TH2 cytokines, suggesting that TH2 adaptive immune responses also play a key role in the development of filarial-induced lymphedema. (Debrah et al., 2006)

Early reports documenting the potential for tetracyclines to treat filariasis were related to the anti-parasitic effects of the drug. (Bandi et al., 1999; Hoerauf et al., 2003) A double-blind, placebo-controlled trial of 18 patients with bancroftian filariasis in Ghana compared lymphedema in patients randomized to a 6-weeks regimen of 200 mg/day doxycycline vs placebo (ISRCTN 14757). (Debrah et al., 2006) All patients were also treated with standard anti-parasitic treatment, and lymphedema outcomes were analyzed 12 and 24 months later. At the 1-year time point, patients treated with Doxycycline had decreased serum levels of VEGF and soluble VEGFR3. These changes correlated with a reduction in the mean stage of lymphedema as measured by improved skin texture and reduced skin folds. However, it is important to note that despite the evidence suggesting that soluble VEGFR3 is related to impaired lymphangiogenesis in preclinical models, this is the first clinical trial to utilize soluble VEGFR3 levels as a clinical outcome measure. (Mäkinen et al., 2001a) These findings led to a larger study on 162 patients with mild to moderate lymphedema randomized to 3 treatment arms comparing a 6-weeks course of treatment with amoxicillin (1000 mg/d), Doxycycline (200 mg/d), or placebo (ISRCTN 90861344). (Mand et al., 2012) At 1 and 2-years follow-up, nearly 44% of patients treated with Doxycycline showed improvements in their disease status. In contrast, improvements were noted in only 3.2 and 5.6% of patients treated with amoxicillin or placebo, leading the authors to recommend a 6-weeks course of doxycycline treatment every year or every other year for patients with mild-to-moderate filarial lymphedema.

The finding that the beneficial effects of Doxycycline may be related to anti-inflammatory rather than anti-microbial activity—supported by the fact that Doxycycline was more effective than amoxicillin—has led to an interesting study using a mouse model of filarial lymphedema. (Furlong-Silva et al., 2021) This model, similar to the clinical scenario, was associated with impaired lymphatic function and increased circulating levels of VEGF-C. In addition, filarial-induced lymphatic dysfunction was dependent on IL-4 receptor immune responses. The study also showed that the beneficial effects of tetracycline in this model were related to decreased monocyte recruitment, inhibition of alternatively activated macrophage polarization, and decreased TH2 differentiation. (Furlong-Silva et al., 2021) With an established safety record in other inflammatory disorders, such as rheumatoid arthritis and rosacea, these findings suggest that doxycycline therapy may be a useful adjunct for the treatment of other forms of lymphedema. (Sapadin and Fleischmajer, 2006)

Despite their promising preclinical and clinical findings, the majority of clinical trials exploring the role of anti-inflammatory agents in lymphedema have many limitations (e.g., small numbers of patients, non-randomized studies, retrospective analyses, heterogenous outcome measures, etc.).

**FIGURE 2:**
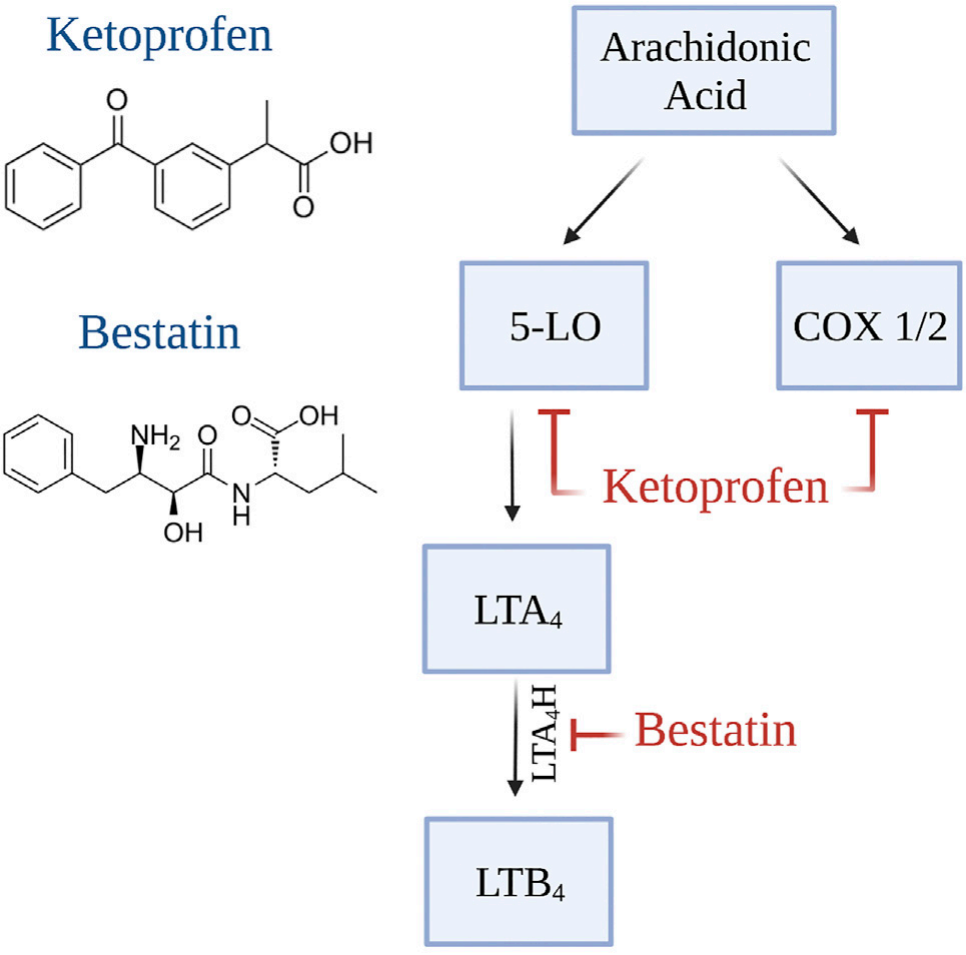
Mechanism of action of LTB_4_ inhibitors (Created with BioRender).

**FIGURE 3:**
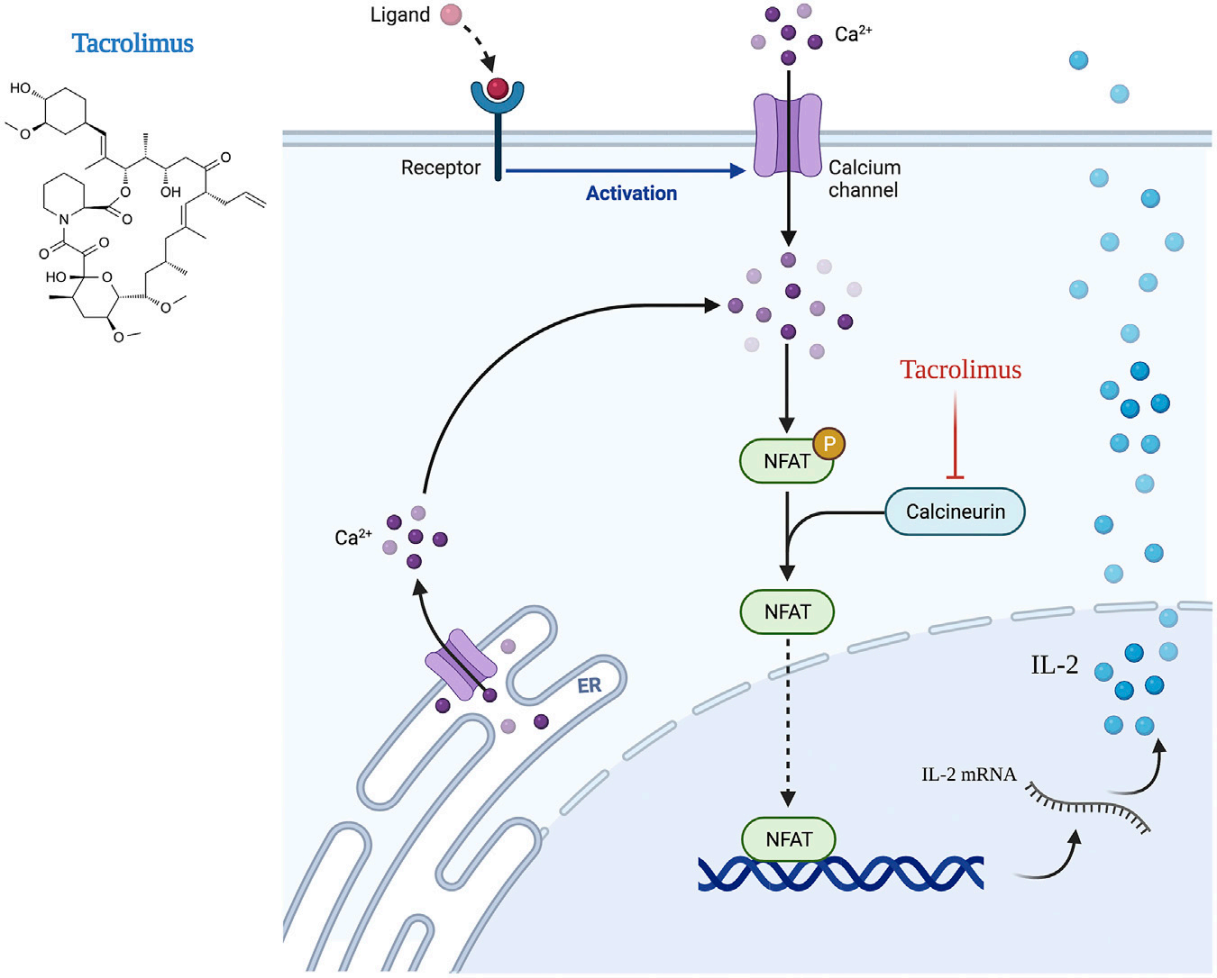
Mechanism of action of tacrolimus (Created with BioRender).

**FIGURE 4:**
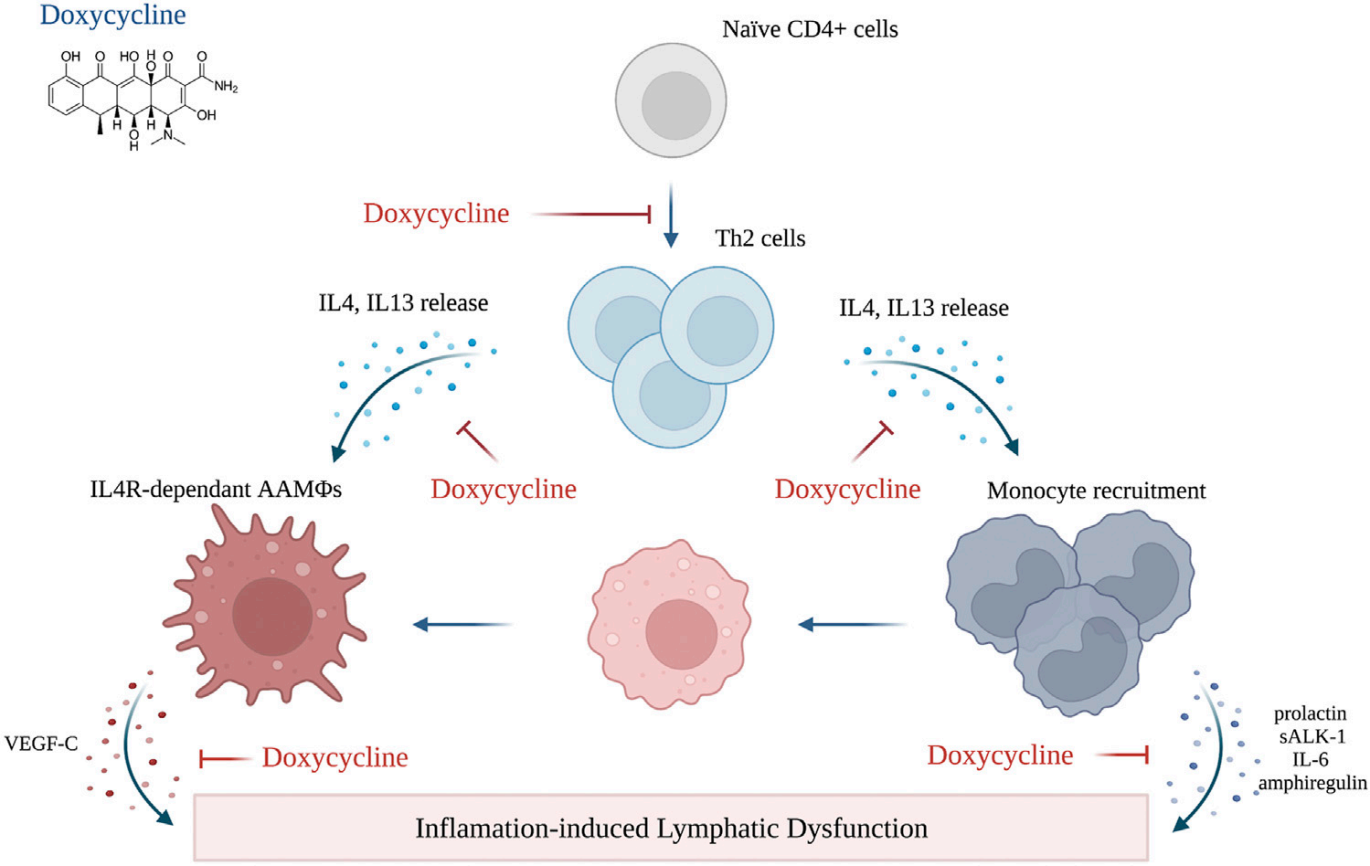
Mechanism of action of doxycycline (created with BioRender). Adopted from: (Furlong-Silva et al., 2021).

Table 2 summarizes the current knowledge regarding preclinical and clinical studies utilizing immune-based agents to treat secondary lymphedema. The mechanism of action of the pharmacological agents reviewed in this section is illustrated in Figure 2, Figure 3, Figure 4.

**TABLE 2:** Anti-inflammatory Treatments for Lymphedema.

*Authors*	Agent	Study Type (sample size if clinical)	Outcomes
Nakamura et al. (2009)	Ketoprofen (NSAID)	Preclinical	Reduced tail volumes, epidermal thickness; improved histology
			Increased expression of TNFa, MCP-1, VEGF-C, VEGFR-3 and Prox-1
Tian et al. (2017)	Bestatin (LTB_4_ inhibitor)	Preclinical	Reversed tail edema, dermal thickening, lymphatic dilatation, and lymphatic permeability
			Increased lymphatic transport rate
Rockson et al. (2018)	Bestatin (LTB_4_ inhibitor)	Clinical (open label trial, *n* = 21; placebo-controlled trial, *n* = 34)	Reduced dermal thickness, collagen thickness, mucin deposition, and perivascular inflammation
			Decreased plasma G-CSF expression
Cribb et al. (2021)	Bestatin (LTB_4_ inhibitor)	Preclinical	Increased lymphatic contractility
Gardenier et al. (2017)	Tacrolimus (T-cell proliferation inhibitor)	Preclinical	Reduced tail volume, fibroadipose deposition, dermal backflow, T cell and macrophage inflammatory response, and production of IFN-g
			Increased collecting vessel pumping and formation of collateral lymphatics
Mehrara et al. (2021)	IL4/IL13 Neutralizing Antibodies	Clinical (*n* = 9)	Reduced skin stiffness, epidermal thickness, skin collagen deposition, infiltration of mast cells and T cells
			Improved QoL outcomes

## Anti-Fibrotic Treatments for Lymphedema

Fibrosis, like inflammation, is a clinical hallmark of lymphedema. (Kataru et al., 2019) Lymphedema biopsy samples demonstrate accumulation of collagen fibers in the reticular and papillary dermis, progressive fibrosis and luminal obstruction of collecting lymphatics, and dense accumulation of collagen fibers around capillary lymphatics. (Mihara et al., 2012) Fibrosis, independent of lymphedema, is associated with decreased lymphatic function in wound healing, radiation, and obesity. (Avraham et al., 2002; Clavin et al., 2008; Zampell et al., 2012b)

Transforming growth factor beta-1 (TGF-B1) is a protein growth factor that plays an essential role in various physiologic and pathologic settings. Numerous clinical and experimental studies have implicated TGF-B1 as an important regulator of pathological fibrosis. (Meng et al., 2016) These effects are related to increased fibroblast collagen deposition, decreased extracellular matrix turnover, and modulation of inflammatory response. (Meng et al., 2016) The expression of TGF-B1 is increased in clinical lymphedema specimens and mouse models of the disease, suggesting that this growth factor also plays a role in regulating lymphedema-induced fibrosis. (Sano et al., 2020) Inhibition of TGF-B1 with a small molecule inhibitor decreases fibrosis and improves radiation-induced lymphatic dysfunction. (Avraham et al., 2010) Other studies have shown that inhibition of TGF-B1 using small molecule inhibitors or neutralizing antibodies decreases fibrosis and the severity of lymphedema in the mouse tail model. (Clavin et al., 2008; Sano et al., 2020; Yoon et al., 2020)

Anti-TGF-B1 treatments also increased collateral lymphatic formation, a finding that is supported by the anti-lymphangiogenic activity of TGF-B1 *in vitro* and some physiologic settings. (Sano et al., 2020) Thus, there is significant potential for anti-fibrotic and anti-TGF-B1 therapies for lymphedema treatment. However, effective means of chronically decreasing TGF-B1 activity without induction of autoimmune responses remains a challenge and will require additional investigation.

## Conclusions

The lack of a cure for lymphedema is largely related to an insufficient understanding of the pathophysiology of the disease. Advances in the last 2 decades have made important inroads in this area and have identified many potential treatment options, including lymphangiogenic cytokine delivery, anti-inflammatory medications, and anti-fibrotic strategies. These treatments may be used in conjunction with traditional methods of treating lymphedema or as adjuncts to surgical management. The possibility of treating lymphedema with topical medications is exciting as this approach may decrease the potential toxicity of systemic treatment. Nevertheless, additional studies with larger sample size, improved methodological quality and standardized and reproducible outcome measures are required to bring these options from the bench to the bedside.
